# Update on PET Radiopharmaceuticals for Imaging Hepatocellular Carcinoma

**DOI:** 10.3390/cancers15071975

**Published:** 2023-03-25

**Authors:** Nozipho Nyakale, Luca Filippi, Colleen Aldous, Mike Sathekge

**Affiliations:** 1 Department of Nuclear Medicine, Sefako Makgatho Health Science University & Dr George Mukhari Academic Hospital, Pretoria 0208, South Africa; 2 Department of Nuclear Medicine, Santa Maria Goretti Hospital, Via Canova 3, 04100 Latina, Italy; 3 Department of Genetics, College of Health Sciences, University of KwaZulu Natal, Durban 4058, South Africa; 4 Department of Nuclear Medicine, University of Pretoria & Steve Biko Academic Hospital, Pretoria 0001, South Africa

**Keywords:** hepatocellular cancer, positron emission tomography, PET, PSMA, Choline, FDG, FAPI, yttrium-90

## Abstract

**Simple Summary:**

Hepatocellular carcinoma (HCC) causes significant mortality globally. The majority of patients experience resistance to systemic therapies and often undergo recurrence or disease progression even after aggressive local therapies. Management of HCC is determined by the extent and stage of the disease using blood tests and imaging tests per the BCLC guidelines. A number of positron emission tomography (PET) radiopharmaceuticals have been investigated with the aim of finding the most appropriate biomarker, which can help to accurately stage the disease to optimise treatment of this cancer. More trials are necessary to determine the accuracy of these PET radiotracers and their impacts on initial staging, assessing the treatment response and determining recurrence early enough to minimise morbidity.

**Abstract:**

Numerous positron emission tomography (PET) targets for detection and staging of hepatocellular cancer have been developed in recent years. Hepatocellular carcinomas (HCCs) are clinically and pathologically heterogeneous tumours with a high tendency to be aggressive and unresponsive to chemotherapy. Early detection is essential, and the need for an adequate imaging biomarker, which can overcome some of the limitations of conventional radiological imaging, is persistent. Flourine-18 (^18^F) flourodeoxyglucose (FDG), the most widely used PET radiopharmaceutical, has proven disappointing as a possible staple in the evaluation of HCC. This disappointment had led to experimentation with carious radiotracers, such as the choline derivatives, acetate, and prostate-specific membrane antigen, which appear to complement and/or enhance the role of FDG. In this study, we look at the various PET radiopharmaceuticals that have been used for imaging HCC and the particular pathways that they target in HCC and liver cancers.

## 1. Introduction

Hepatocellular carcinomas (HCCs) are tumours of the parenchymal cells of the liver arising from the malignant transformation of hepatocytes [1]. Approximately 75–80% of liver cancers are histologically hepatocellular carcinoma globally, and these tumours are responsible for the high rate of cancer deaths worldwide, with a low overall survival rate [2,3,4]. 

Hepatitis B virus (HBV), a DNA virus, is responsible for 75% to 80% of virus associated HCC, whereas hepatitis C virus (HCV) is responsible for 10% to 20% [2,4,5]. HBV infection promotes mutations in liver cells by inducing a chronic necro-inflammatory disease, subsequently leading to HCC [4]. The lifetime risk for developing HCC in HBV-infected individuals ranges from 10% to 25%. This risk is thought to be exacerbated by co-factors, such as demographics; and by viral factors, such as high HBV replication levels, HBV genotype, infection duration, and coinfection with HCV or human immunodeficiency virus (HIV). Chronic HCV infection increased risk by 10- to 20-fold, but unlike HBV, which is known to integrate into the host genome, HCV is unlikely to initiate tumourigenesis [4]. Major risk factors for HCC vary in different regions with chronic aflatoxin B1 (AFB1) infection and aflatoxin B1 (AFB1) exposure noted to be the major risk factors in some, while HCV infection, excessive alcohol consumption, and diabetes/obesity/metabolic syndrome play more important roles in others [2].

These tumours rely on new vessel formation for growth, hence their functional and structural abnormalities. This angiogenesis causes destabilisation of the existing microvasculature, causing vascular hyperpermeability and thus leaky vessels, remodelling of the extracellular matrix, and endothelial cell activation. HCCs are therefore clinically and pathologically heterogeneous, often with hypovascular areas, hypoxia, and necrosis, causing non-uniform blood flow and heterogeneous delivery of chemotherapeutic drugs and oxygen [1]. The dual blood supply of the liver also results in the housing of an abnormal environment prone to accommodating more aggressive malignancies [1]. They may present as a single, well-demarcated mass that progresses to a single large lesion or as multifocal, infiltrative tumours with a high propensity towards local, regional, or distant metastases [1,6].

HCC most frequently metastasises to the regional lymph nodes, lungs, and bones. The majority of patients present with already advanced-stage malignancy when patients have abdominal pain or hepatic decompensation, and they often have associated cirrhosis and thus limited treatment options [7]. These tumours are also prone to progression, resistance to therapy, and recurrence despite aggressive local therapy at initial presentation [6]. 

Mortality from HCC continues to rise worldwide, indicating the need for improvement in the work-up, classification, and staging systems to adequately guide effective treatment choices [6]. 

Individualised treatment selection based on each patient’s unique clinical and molecular phenotype forms an important part of the staging of HCC, but it remains challenging due to the heterogeneity of the disease.

Available treatment options include liver transplantation, partial liver resection, and ablation, which offer a high rate of complete response dependent on early detection and management [3]. These treatment options are generally reserved for early-stage disease (Barcelona Clinic Liver Cancer (BCLC) stage 0 to stage A). Intermediate and advanced-stage disease (BCLC Stage B and C) may receive systemic therapy, transarterial chemoembolisation (TACE), and radioembolisation (i.e., selective internal radiation therapy [SIRT]), while patients with end-stage disease (BCLC stage D) can only receive palliative care [8]. More novel therapies have changed the landscape of treatment of HCC. Multi-kinase inhibitors, such as sorafenib, targeting vascular endothelial growth factor receptors 1–3, which have been the standard of care, have in some cases been replaced by immunotherapy plus anti-VEGF combination regimens. Novel anti-angiogenic factors, such as cabozantinib, may be used as a second-line treatment option [9].

Unfortunately, up to 50% of patients who are able to undergo curative resection still develop intrahepatic recurrence from second primaries or from intrahepatic spread [5,6]. The prognosis of HCC is therefore partly dependant on the stage at presentation of the patient. When detected at an early stage, it opens up the possibility of curative, life-prolonging treatment [10].

Diagnosis of hepatocellular cancer is variable and may be based on pathology or determined non-invasively using a combination of blood tests and imaging tests [11]. The use of tumour markers, such as AFP, and the number of required imaging modalities indicated remain uncertain [11,12]. 

The classification systems of HCC also vary, and their use may be subject to geographic variability. They may be based on clinical or imaging findings (i.e., clinical) or even on findings of open surgical resection or exploration (i.e., pathologic) [6].

Patients who do not receive liver transplantation are noted to be at a high risk for tumour recurrence, indicating a critical need for close interval follow-up during therapy for the early detection of tumour recurrence when treatments are more effective, and the amount of injury to benign liver tissue is minimal. Unfortunately, for more aggressive biology, tumours with the initial appearance of early-stage disease may recur with multifocal or diffusely infiltrating disease that is difficult to treat [6].

The ultimate objective of classification and staging methods is to be able to select the best treatment options for patients. This objective remains challenging in practice because of the variability noted, and many centres do not treat strictly according to the available guidelines [6].

## 2. Role of Imaging

Imaging in HCC is necessary for the early detection of these tumours, differentiation from regenerative nodules and other benign liver tumours, accurate staging, and identification of recurrence, as well as in response to therapy evaluation. Contrast-enhanced computed tomography (ceCT) of the chest, abdomen, and pelvis or magnetic resonance imaging (MRI) is often required for intrahepatic staging of HCC. Contrast enhanced CT is regarded as the preferred imaging modality for more reliable detection of intrahepatic and extrahepatic tumour metastases.

## 3. Conventional Radiological Imaging

Conventional imaging traditionally includes transabdominal ultrasound (US), ceCT, and/or MRI in the screening and evaluation of hepatic lesions. These modalities involve evaluation of the structural aspects of the malignancy and greatly rely on size as a major determinant in test sensitivity. The majority of HCC patients have underlying pathologies, such as chronic hepatitis and cirrhosis, causing structural distortion, and other liver pathologies, such as haemangiomas and regenerative nodules, may decrease the effectiveness of structural imaging.

Ultrasound is the initial imaging test suggested by all guidelines since it is able to detect small lesions and is readily available. Ultrasound is also used for screening and has been reported to have a sensitivity of between 65% and 80% and specificity of more than 90% [13]. Contrast enhanced ultrasound is also useful for the detection and differentiation of portal vein thrombosis from tumour-in-vein in this condition [14]. Unfortunately, it is operator dependent and may cause difficulty in identifying lesions in a nodular cirrhotic liver [13]. 

Contrast-enhanced multiphasic CT and MRI remain the mainstay for diagnosis and staging of HCC and currently serve as the gold standard in this disease. Surveillance is often performed with serial dynamic multiphasic CT or MRI [5,15,16].

In clinical practice, the appropriate management of a given specific stage of HCC depends on the accuracy of the imaging diagnosis of HCC. The imaging diagnosis of HCC is characterised by “arterial phase hyperenhancement” and “washout” after intravenous contrast administration because HCC only contains arterial blood that retains contrast, whereas the rest of the liver maintains its dual blood supply from both the hepatic artery and the portal vein. These findings provide specificity of 87% to 95% for the diagnosis of HCC [15]. Imaging allows for non-invasive diagnosis of HCC and for treatment to be initiated without confirmatory biopsy as long as typical imaging characteristics for HCC are present. The rationale is based on the pre-test probability of HCC being sufficiently high in patients with cirrhosis and the pre-test probability of lesions that may mimic HCC on imaging being low [17]. The Liver Imaging Reporting and Data System (LI-RADS) is incorporated to standardise reporting and care in patients with or at risk for HCC with regard to surveillance with US; diagnosis with CT, MRI, or contrast material–enhanced US; and assessment of treatment response with CT or MRI. This system takes into account various algorithms that consider factors such as the dual supply of the liver and the tumour enhancement and washout in relation to the arterial and portal venous phases to classify nodules as HCC for diagnosis and follow-up [18]. 

A meta-analysis published in 2015 by Lee and co-workers evaluated the diagnostic accuracy of ceCT and MRI for HCC. They showed that MRI is generally more sensitive than ceCT with sensitivity of 79% vs. 72%, respectively, on a per-lesion basis and more significant differences in a head-to-head comparison of studies with paired diagnostic accuracy data for MRI and ceCT (80% vs. 68%, *p* = 0.0023) [16].

The presence of arterial uptake followed by washout is highly specific for HCC, but a false-negative rate of 20% to 38% has been reported in the use of MRI for the diagnosis of small HCC lesions between 1 and 2 cm in size with atypical features [19]. Thus, with regard to tumour size, studies have shown markedly decreased sensitivity estimates for subcentimetre HCCs compared with lesions 1 cm or larger. The per-lesion sensitivity estimate was even lower for ceCT (31%) compared with MRI (48%) in subcentimetre lesions [16].

Advanced liver cirrhosis, with more severe morphologic distortions of segments and greater numbers of benign cirrhosis-related nodules, are commonly associated with HCC, but disease severity is known to affect the sensitivity of conventional imaging due to changes in liver structures resulting from disease [16]. In many cases, it is also difficult to detect extrahepatic secondaries in anatomical tests, such as identifying a disease in normal-size lymph nodes or early skeletal involvement, thus limiting the accurate staging of the disease [16].

Molecular imaging using PET/CT has also been studied in an attempt to improve the staging and follow-up of patients. PET-based imaging with the commonly used ^18^F FDG tracer is thought to be inaccurate in evaluating early tumours. It is preferred in more advanced disease and has been incorporated in some tertiary institutions in exceptional cases due to the complimentary nature suggested in the literature.

## 4. PET/CT Imaging of HCC

Positron emission tomography (PET) is an imaging modality used in the evaluation of various malignancies by incorporating the use of radiolabelled molecular targets. This modality is functional imaging based on the ability to detect changes in the properties of the tissue that occur before structural changes are seen [14]. Whole body PET scans detect the tumour cells’ metabolic activity and viability, the presence of proteins or receptors that characterise the cell, the cancer cells’ aggressiveness, and the extent of spread beyond the primary site. Hybrid imaging incorporating conventional imaging with CT or MRI on the same device as the PET scanner further increases the sensitivity and specificity of PET scans [14,20].

A number of PET radiotracers have been evaluated for their potential role in imaging HCC.

### 4.1. Fluorine-18-fluoro-2-deoxyglucose

Fluorine-18-fluoro-2-deoxyglucose (^18^F -FDG or FDG) is the most commonly used PET radiotracer in oncology. It is a glucose analogue formed by replacing the hydroxyl group at the C2 position in the glucose molecule with ^18^F. The half-life of ^18^F (110 min), compared with that of the other positron emitters, allows for more practical use and access for centres that may not have direct access to an onsite cyclotron [21,22]. 

Glucose metabolism is noted to be increased in most tumour cells due to increased levels of glucose transporter proteins and levels of intracellular enzymes that promote glycolysis, such as hexokinase and phosphofructokinase. FDG competes with glucose at transport sites on the cell membrane and in a variety of these intracellular enzymatic pathways. In HCC, increased ^18^F FDG transport into cancer cells has been associated with GLUT1, GLUT3, and GLUT12 receptors [3,7]. In most malignant cells, relatively low levels of glucose-6-phosphatase lead to accumulation and trapping of FDG intracellularly as 2-fluoro-2-deoxyglucose-6-phosphate (FDG-6-P0_4_) due to phosphorylation by the enzyme hexokinase (HK), which phosphorylates glucose in the glycolytic pathway and allows for the visualisation of increased FDG uptake compared with that of normal cells [7,23]. The enzymatic activity of well-differentiated HCC may resemble normal hepatocytes and hence tend to have high glucose-6-phosphatase activity, allowing for dephosphorylation of intracellular FDG and its resultant egress from the cells. A decrease in differentiation increases glycolytic enzymes, decreasing glucose-6-phosphatase activity and causing a significant increase in the kinetic rate constants, and uptake values are higher in poorly differentiated HCC [7]. 

In HCC, the varying degrees of activity of glucose-6-phosphatase and glucose transporters, as well as the degree of differentiation, result in variable accumulation of FDG [21,24,25], thus limiting the diagnostic accuracy of FDG PET in the evaluation of HCC.

#### 4.1.1. Intrahepatic Detection

Torizuka et al. used FDG-PET to quantitatively evaluate the glucose metabolism of HCC compared with in vitro enzymatic activity of glucose metabolism and histologic grading of HCC. This study revealed that hexokinase activity was significantly higher in high-grade HCC than in low-grade HCC, the glucose-6-phosphatase activity of high-grade HCC tended to be lower than in low-grade type HCCs, and FDG uptake measured by the standard uptake value (SUV) was significantly higher in high-grade HCC than in low-grade HCC [24]. FDG PET may be useful in estimating the enzymatic activity and, therefore, the histologic grade of HCC. 

Normal liver tissue has lower levels of hexokinase and high levels of glucose-6-phophatase, thus resulting in lower accumulation and trapping of FDG-6-phosphatase in comparison to HCC cells, which have lower levels of this enzyme. However, the high rate of gluconeogenesis of well-differentiated HCC may be comparable to that of normal liver tissue and results in ^18^F FDG uptake, which is similar and thus difficult to differentiate between the two [7,26]. Some studies could not confirm the finding that FDG uptake correlated more with poorly differentiated tumours and showed that positive findings were evenly distributed between different classes of differentiation [27]. The heterogeneity of HCCs thus leads to a notable rate of false-negative studies, with an overall sensitivity of FDG PET/CT in detecting HCC reported to range between 50% and 70% [25,27,28,29].

To date, FDG PET has demonstrated limited consistency in use as an imaging tool in patients at risk for HCC, affecting its popularity in the evaluation of intrahepatic disease [21,30]. 

A study that evaluated the ability of FDG to distinguish between benign and malignant lesions in patients with clinically pathological liver lesions, which would allow potentially for resection of these lesions, demonstrated that up to 30% of HCC lesions did not accumulate FDG avidly and thus indicated a limitation in using this radiotracer to classify these lesions accordingly [29]. 

FDG assessment was performed of tumours ranging from 1.5 to 20 cm in size with a mean diameter of 5.7 cm, with varying diagnoses, including liver cirrhosis, anti-HCV and HBsAg sero-positive, and subsequent histological or cytological diagnosis of HCC. Of these cases, 55% of tumours demonstrated FDG accumulation with varying degrees of uptake greater than normal liver uptake, while 45% were equal to and less than liver uptake and were concluded to be FDG negative for HCC [7]. In this cohort, it was noted that higher FDG uptake was prominent in less-differentiated tumours, and it appeared to have a lower sensitivity for detecting HCC compared to CT scans [7]. FDG PET was significantly more sensitive if the size of the tumour was larger, i.e., >5 cm in diameter, than for those <4 or 5 cm in size, and the FDG detection sensitivity of primary HCC increased with increasing tumour size [27,31]. 

Park et al. also showed significant association of FDG uptake with high serum α-fetoprotein, modified UICC stage, BCLC stage, larger tumour size, number of tumours, and the presence of portal vein invasion [32].

A correlation of FDG uptake with the level of alpha fetoprotein (AFP), a well-known protein tumour marker of HCC, was indicated and thought to occur because of increased tumour growth and metabolic activity causing increased AFP production and increased glucose uptake [27,31]. Increased AFP levels are noted in more aggressive cases of HCC. 

FDG PET offers no advantages over currently available imaging modalities in the setting of small-foci HCC.

Shiomi et al. also showed that the tumour-to-non-tumour SUV ratios in livers with HCC tumours correlated with tumour volume-doubling time (r = −0.582; *p* = 0.006) and could be predictive of cumulative survival rate based on the SUV ratio. A significantly lower cumulative survival rate in the group with a SUV ratio of 1.5 or less was evident, compared to those whose SUV ratios were greater than 1.5 [33].

#### 4.1.2. Metastases

Although the sensitivity of FDG PET/CT in evaluating soft tissue and lymph node extrahepatic metastases was not more impressive that of than ceCT in a study by Kawaoka et al., it demonstrated higher sensitivity for the detection of bone metastases compared with ceCT and bone scintigraphy. It showed mean sensitivity and specificity for diagnosis of bone metastasis of 83.3% and 86.1% for FDG, respectively, versus 41.6% and 94.5% for ceCT and 52.7% and 83.3% for bone scintigraphy. FDG PET/CT changed the TNM stage in four cases by detecting additional lymph nodes and bone metastases than conventional imaging [34]. In the study by Khan et al., although FDG PET underperformed in the detection of the primary lesions in comparison with conventional imaging with CT, it was able to detect metastases in three patients that were not seen on abdominal CT scans. FDG PET’s sensitivity in the detection of extrahepatic metastases was higher compared with CT scans, 80% vs. 60%, respectively, and therefore had a significant impact on determining management and eligibility for transplantation as a treatment option [27]. 

FDG is therefore thought to play a rather complementary role to CT imaging and has been recommended as part of the staging and management of selected patients with HCC [5,35]. FDG PET was useful in the detection of extrahepatic spread in patients who demonstrated uptake and in some cases was able to detect unsuspected areas of HCC spread [30].

The clinically significant impact of FDG PET/CT in HCC appears to rather lie in its ability to guide biopsy of necrotic tumours, identify distant metastases, and monitor response to treatment with regional therapy, as well as detect recurrence [35,36]. FDG-PET seems to further reflect the degree of differentiation and thus the prognosis of HCC.

Poorly differentiated HCCs, which are more likely to metastasize, tend to be FDG avid compared to the well-differentiated types. Extrahepatic metastases were identified with high sensitivity by FDG PET/CT. Hence, FDG uptake in HCC acts as a marker of differentiation with the phosphorylation constant (k3) and SUV of high-grade HCCs significantly more avid than those of low-grade HCCs as demonstrated by Shiomi et al. SUVs can therefore provide insight into the histopathologic nature and thus prognosis of the tumour [24,33].

FDG PET/CT appears to provide insight into the extent of the metabolic activity of the tumour and, as a result, in predicting outcome. As a result of the mixed utility and variable sensitivity shown by FDG PET/CT in the detection of HCCs, efforts to seek alternate tracers for screening patients at risk, staging, and monitoring regional therapy of patients with HCC continue. 

### 4.2. Gallium-68 PSMA

Prostate-specific membrane antigen (PSMA) is a transmembrane glycoprotein highly expressed on prostate adenocarcinomas and has been vigorously targeted for both clinical imaging and targeted therapy. Gallium-68 (^68^Ga)-labelled PSMA is a safe, non-invasive imaging tracer commonly used in clinical imaging of prostate cancer and has demonstrated superior performance in localised and metastatic disease, as well as in early detection of disease recurrence compared to conventional imaging [37,38,39]. 

Although previously thought to be specific to prostate cancer, multiple reports have demonstrated avidity of radiolabelled PSMA and its potential role in HCC [40,41,42]. 

Early and more advanced HCC may be characterised by neovascularisation through the development of arteries, which become the dominant blood supply of the tumour. The mechanism of uptake of PSMA is believed to be related to the characteristic nature of neovascularisation, which forms a significant feature in the diagnosis of HCC [16,43]. Further evidence has suggested that PSMA, found in the neovasculature of many tumours, may play a role in the regulation of angiogenesis; however, the mechanism of this process is unclear [44].

Denmeade et al. demonstrated PSMA expression in tumour-associated endothelial cells in the majority of HCCs, indicating the possibility of avidity with this peptide in imaging, as well as targeted tumour therapy settings. Positive staining could be demonstrated in 95% of hepatocellular and renal cancers [43]. 

In a study by Lee et al., vascular endothelial PSMA expression was demonstrated in the HCC tumour associated vascular endothelium of a woodchuck model bearing spontaneous HCC. Sergeva et al., who demonstrated steady uptake even in progressive disease, further validated this [16,40,45].

**Figure 1:**
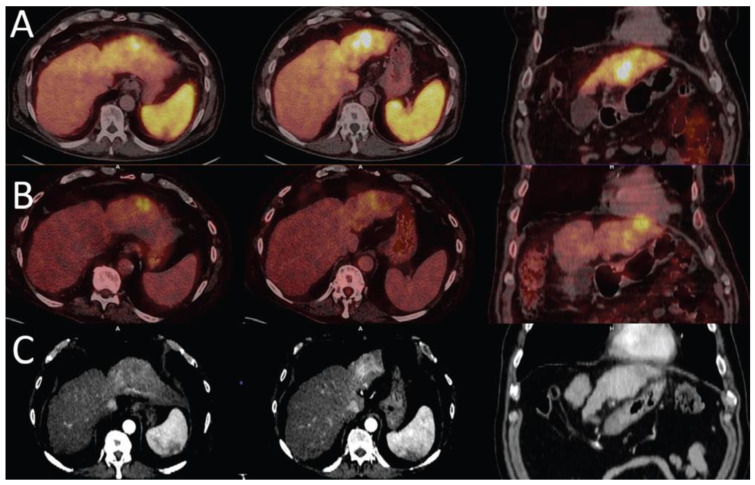
Liver cirrhosis with HCC in the left lobe of the liver. ^68^Ga PSMA (**A**) shows higher uptake in the hyperenhancement areas of the arterial phase CT images (**C**) than the focal ^18^F -FDG uptake area (**B**), which is noted to appear hypodense on CT (images reproduced with permission from [46]).

Tumour viability is essential in the diagnosis, staging, and management of HCC. A molecular imaging agent, which detects the majority of HCC tumours, may assist in more accurate staging of the disease and evaluation of treatment response. The limitations of FDG PET/CT in the imaging of primary HCC thus justify the need for a tracer that may be more reliable and appropriate for adequate management of patients. The surfacing reports indicate the possibility of this role for ^68^Ga PSMA in imaging of HCC (Figure 1) with possible additional application to PSMA-based radionuclide therapy in HCC.

Angiogenesis has been demonstrated to contribute to progression of a number of cancers, but the actual angiogenic processes do not appear to be regulated by the same signals and often present distinct pathologies. This fact indicates that ^68^Ga PSMA PET/CT imaging may play a role in the assessment of treatment response and the selection of those patients with hepatocellular carcinoma who may benefit from non-PSMA-targeting antiangiogenic treatment strategies. 

#### 4.2.1. Intrahepatic Detection

With the known shortcomings of conventional imaging of HCC especially in patients with cirrhosis, ^68^Ga PSMA imaging may be able to assist in the differentiation of benign versus malignant lesions. No significant difference was evident between the uptake of ^68^Ga PSMA when comparing cirrhotic and non-cirrhotic livers [42,47]. PET/CT with ^68^Ga PSMA could detect subcentimetre lesions with a diameter of 8 mm that had been described on MRI [46]. 

In the study by Kesler et al., ^68^Ga-PSMA PET/CT was positive in all liver lesions that fit the criteria for HCC on radiologic imaging, with the exception of a single lesion that on ceCT was seen to have only peripheral contrast enhancement. The ^68^Ga PSMA positive tumours that underwent immunohistochemistry showed intense intratumoral microvessel staining for PSMA, showing no staining demonstrable on the epithelial tumours, consistent with the theory that PSMA plays a major role in angiogenesis regulation [42]. The pattern of uptake of ^68^Ga PSMA also correlated with that of ceCT, in which certain tumours, which either showed dedifferentiation or had areas of necrosis that developed due to excessive tumour growth, showed less arterial enhancement, as well as reduced PSMA uptake, resulting in a mosaic heterogeneous uptake pattern [42].

Hirmas et al. reported comparable accuracy of 97% for CT and ^68^Ga PSMA (sensitivity of 97%, specificity and positive predictive value of 100%, and negative predictive value of 80%) in whole-liver HCC evaluation, including in patients with liver cirrhosis [47]. 

#### 4.2.2. Metastases

Kesler et al. also demonstrated increased ^68^Ga PSMA in metastatic lesions in bone marrow, adrenal glands, and an abdominal implant that was missed on conventional imaging [42]. Similarly, ^68^Ga PSMA PET was able to detect more distant metastatic lesions than CT (13 versus 9) in the study by Hirmas et al. [47]. The lesions that were missed on CT or deemed as non-pathological were in the skeletal system and mediastinal lymph nodes. The accuracy, sensitivity, specificity, positive predictive value, and negative predictive value were 100% for ^68^Ga PSMA PET in the detection of metastatic lesions. In this instance, although CT demonstrated similar specificity and positive predictive value to those of ^68^Ga PSMA PET, it had lower sensitivity of 67% and slightly lower accuracy and negative predictive value of 97% and 93%, respectively [47]. Overall, ^68^Ga PSMA PET/CT resulted in a change in management in up to 47.5% of patients. 

### 4.3. Radiolabelled Choline Derivatives

Choline is a component of phosphatidylcholine, which is an essential element of cell membrane phospholipids. Radiolabelled choline is internalised in the cells by facilitative transport and passive diffusion; it is then phosphorylated in the cytoplasm to form phosphorylcholine and finally incorporated in the cell membrane [48,49]. Choline can be radiolabelled using ^11^C or ^18^F for PET/CT imaging. ^18^F Fluorocholine (FCH) is a choline analogue that behaves as a substrate for choline kinase and thus is involved in the initial steps of choline metabolism, leading to phosphatidylcholine synthesis [50]. In liver cancer, there appears to be upregulation of the cytidine diphosphate (CDP) choline pathway, which produces phosphatidylcholine from choline in support of tumour cell proliferation. ^18^F fluorocholine thus localizes in HCC, possibly due to the high choline content of this cancer as a result of increased proliferation and metabolism compared to normal liver tissue [26,50,51]. 

The main advantage of ^18^F Fluorocholine over its ^11^carbon-labelled counterpart is that is more readily available due to its longer half-life compared to the short 20-minute half-life of ^11^carbon, which requires an on-site cyclotron. ^11^C Choline has its own advantage over FCH since only 2% of the injected dose is excreted in the urine; therefore, it allows for better evaluation of pelvic disease, and its short half-life means less radiation exposure to the patients [52,53].

#### 4.3.1. Intrahepatic Detection

**Figure 2:**
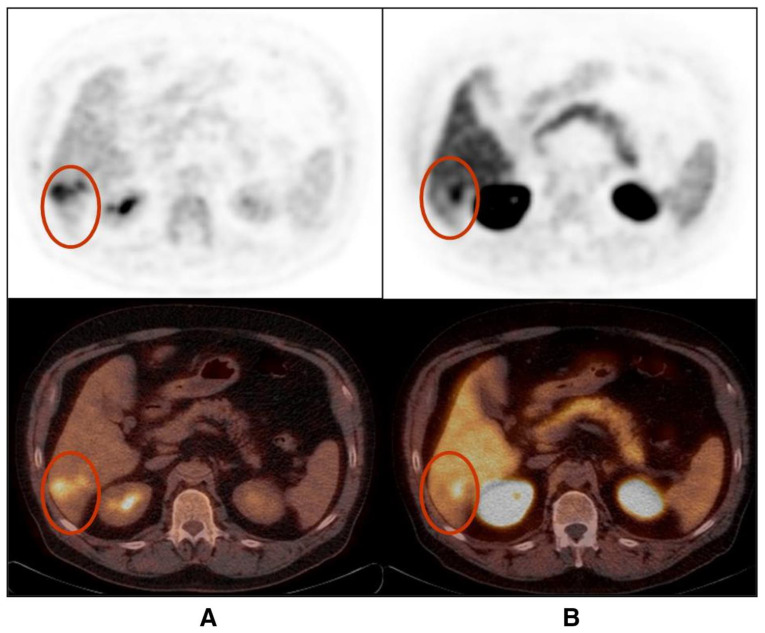
Poorly differentiated hepatocellular carcinoma shows partially congruent uptake of FDG (**A**) and FCH (**B**) (lesions shown in red circles). The FDG-positive areas appear photopeanic on FCH PET/CT (images reproduced with permission from [53]).

In a small prospective study by Talbot et al., FCH was shown to have a detection rate of up to 100% in primary and recurrent HCC tumours with a median signal-to-noise ratio of 1.5 ± 0.38 and with a tendency towards higher uptake in well-differentiated tumours [54]. Yamamoto et al. reported an overall detection rate of 63% with ^11^C-choline PET, which is noted to be higher than the 50% for ^18^F FDG PET, and it was improved for ^11^C Choline in moderately differentiated HCC lesions (75% for FCH vs. 25% for FDG), while the poorly differentiated type exhibited the opposite behaviour (Figure 2) on FDG imaging (42% for FCH and 75 % for FDG) [48].

The proclivity of choline for detecting well-differentiated HCC was further demonstrated, resulting in sensitivity of 94% for FCH compared to 59% for FDG. This outcome was not significantly deterred by the lesion size, and FCH was better able to detect subcentimetre HCC lesions with sensitivity of 88% compared to FDG [26]. FCH was also superior in visualizing smaller subcentimetre lesions compared to ^11^C-acetate [32,33].

In the meta-analysis by Bertagna et al., which included 115 patients from five studies, radiolabelled choline tracers had a pooled detection rate of 84% with a trend towards a higher detection rate when poorly differentiated histology was excluded [51]. Despite this tendency towards better detection of the well=differentiated cancer types, the study by Talbot et al. did not show significantly different sensitivity of FCH and FDG PET/CT in less differentiated HCC, reporting a site-based sensitivity of 76% for FCH vs. 74% for FDG. The authors therefore concluded that these radiotracers were not well suited for accurate non-invasive individual determination of HCC lesion differentiation [26]. FDG uptake has also been noted to be positive in patients with recurrence and higher AFP [55].

In the diagnostic stage of the disease, to characterise liver nodules for the detection of HCC in patients with cirrhosis or chronic liver disease, FCH showed a high false positive rate, accumulating in 88% of focal nodular hyperplasia (FNH) lesions. The false positive rate was lower in cholangitis and in hepatic adenomas. The patient-based specificity of FCH was therefore found to be low in comparison to that of FDG: 47% vs. 94%, respectively [26].

Dual tracer imaging using choline analogues and FDG detects more lesions compared to single tracer imaging. This method significantly increased the sensitivity of PET/CT in the detection of HCC and resulted in a change in management by changing the BCLC staging of the patients. Dual tracer PET/CT imaging with FDG and ^11^C-choline increased the detection rate of HCC, from 63% using FDG PET/CT alone to 89% when both PET tracers were used [56].

The value of choline in monitoring treatment response in patients subjected to selective internal radio-embolisation was also shown to be a promising application in patients with locally advanced, but non-metastatic, HCC and initially elevated AFP [57]. Post-operative recurrence of HCC in the liver is also better detected with FCH in comparison with FDG [58].

Choline may possibly be superior to both acetate and FDG in the detection of HCC, as demonstrated in a hepatitis viral infection-induced woodchuck model of HCC in which ^11^C-choline PET detected all HCCs, surpassing FDG, which detected seven of 13 tumours, and ^11^C-acetate which detected 16 of 17 HCCs [59].

#### 4.3.2. Metastases

Bieze et al. demonstrated extrahepatic metastases, and in their study, the non-HCC lesions were FCH negative, reporting specificity for both hepatic and non-hepatic HCCs of 100%. The same study was able to show the impact of FCH PET/CT on the management of patients, leading to a change in management by revealing additional lesions in 58.6% of the patient population [60]. FCH detected lung and bone metastases with a more intense signal than that of FDG in two patients with extrahepatic disease in the pilot study conducted by Talbot et al., and in the follow up study, the detection of unexpected metastases led to a 7% change in patient management [26,54].

### 4.4. ^11^C-Acetate

PET tracers of lipid metabolism have been proposed for the detection of HCC. Acetate is a metabolic substrate of β-oxidation and a precursor of fatty acid and sterol synthesis. A number of metabolic pathways have been described for the incorporation of acetate in the cells. These pathways include entering the Krebs cycle from acetyl coenzyme A (CoA), as a precursor to β-oxidation through esterification, to form acetyl CoA in fatty acid synthesis by combining with glycine in haeme synthesis or through citrate for cholesterol synthesis. In liver tumours, ^11^C acetate is thought to be a precursor of phosphotidylcholine membrane synthesis, thus taken up mostly in the process of fatty acid synthesis [5,33,49].

#### 4.4.1. Intrahepatic Detection

Ho et al. reported that ^11^C acetate had sensitivity of 87.3% with a lesion-to-normal uptake ratio of 1.96 ± 0.63 in patients with disease localised within the hepatic parenchyma, while the detection sensitivity for ^18^F FDG was only 47.3% in comparison [5]. ^18^F-FDG and ^11^C acetate were found to be complementary in the detection of liver lesions, showing increased metabolism of both these radiotracers and, in some, different parts of the same lesion, accumulated different radiotracers respectively [61]. Histopathological correlation suggested that acetate accumulated in well-differentiated tumours while FDG, as previously described, has a predilection for the more advanced and/or poorly differentiated subtypes [5].

Park et al. further showed significant associations of FDG uptake with high serum α-fetoprotein, modified UICC stage, BCLC stage, larger tumour size, number of tumours, and the presence of portal vein invasion [33]. In contrast, acetate uptake was not associated with clinical factors or staging. Acetate detection sensitivity in primary HCC improved with increasing tumour size, reporting 31.8%, 78.2%, and 95.2% in index lesions of sizes 1–2 cm, 2–5 cm, and 5 cm or more, respectively [33].

Unfortunately, ^11^C acetate PET may have a shortcoming in characterizing hepatic lesions as either benign or malignant, as demonstrated in a case with false-positive intense ^11^C acetate and negative FDG uptake in a lesion histologically confirmed to be a benign angiomyolipoma [62].

Table 1 summarizes how the various PET radiopharmaceuticals performed in comparison with FDG.

#### 4.4.2. Metastases

^18^F FDG outperformed ^11^C-acetate in the detection of vascular and extrahepatic metastases, revealing more lesions than ^11^C acetate [5]. The overall sensitivity of acetate in the detection of metastases was 77% for acetate vs. 85.7% for ^18^F FDG when compared, and in this study, all lesions with acetate uptake were also FDG avid [33]. Therefore, with regard to PET detection of distant metastases of HCC, a trend towards better sensitivity for ^18^F FDG than for ^11^C acetate has been reported. The diagnostic performance was further improved by performing PET/CT examinations with both radiopharmaceuticals [5,33]. The difference in sensitivity between ^11^C acetate and ^18^F FDG in uptake in metastases was not correlated with metastatic tumour size, location, or tumour differentiation [33]. When compared to CT imaging, the dual tracer method was found to be significantly superior, showing sensitivity and specificity of 96.8% and 91.7%, respectively, compared to the sensitivity and specificity of CT imaging of 41.9% and 33%, respectively, in pre-transplant work-up patients [63].

The dual tracer method showed improved detection in response to treatment assessment of trans-arterial chemoembolisation (TACE), compared to individual evaluation with either ^11^C-acetate or ^18^F FDG. This method was not significantly affected by cirrhotic changes in the liver and could be a good predictor of response in liver transplant candidates [63,64].

### 4.5. Fibroblast Activation Protein Inhibitor

Cancer-associated fibroblasts (CAFs) are stromal components abundant in the tumour microenvironment of solid tumours. CAFs are important for tumour cell proliferation, aggressiveness, and migration by secreting and interacting with an array of growth factors and cytokines, leading to overexpression of FAP in stromal and cancer cells [68,71]. Fibroblast activation protein (FAP) is a cell surface glycoprotein commonly expressed in activated stromal fibroblasts of some epithelial tumours [67]. FAP expression is activated in hepatic stellate cells and is found to occur in liver cirrhosis, correlating with the burden of fibrosis in the liver. Fibroblast activation, proliferation, and accumulation are hypothesised to be associated with aggressive tumour behaviour in the liver microenvironment, and the majority of HCCs occur in the background of liver fibrosis and cirrhosis [68,70].

CAF-targeting tracers have been developed based on FAP-specific inhibitors. These radiolabelled FAP inhibitors (FAPIs) have high sensitivity for the detection of liver malignancies due to their high uptake with lower background activity in the normal liver parenchyma [67,68,70]. CAF-targeting tracers also show less heterogeneous uptake compared to other tracers in tumours such as HCC, including in the early disease stages [68].

#### 4.5.1. Intrahepatic Detection

Unlike in normal liver parenchyma, FAPI uptake in cirrhotic and fibrotic liver parenchyma is increased. This phenomenon is caused by the activation, proliferation, and accumulation of fibroblasts in these entities. Up to 80–90% of HCCs are associated with liver cirrhosis and fibrosis and tend to have a relatively lower tumour-to-background ratio (TBR) compared to other tumours in the liver [69,70]. Despite this finding, the different liver cancers did not show significant differences in the intensity of ^68^Ga FAPI uptake [68]. 

FAPI has higher sensitivity in the detection of intrahepatic lesions, missing only five of the total of 35 HCC lesions, translating into sensitivity of 85.7% in comparison to FDG’s 57.1% in a prospective study by Wang et al. [67]. Shi et al. reported 100% sensitivity and 100% specificity in patients with histologically or radiologically confirmed HCC, and although FDG reported similar specificity, it only managed to show sensitivity of 58.8%, similar to that reported by Wang et al. [67,69]. Siripongsatian et al. had the same results showing sensitivity of 100% and 58% for FAPI and FDG, respectively, but FAPI showed some false-positive results in HCC patients [72]. Guo et al. again reported 100% sensitivity specifically for intrahepatic HCC, which was the same for FDG and MRI and only slightly lower for ceCT [70]. The study by Zhang et al. demonstrated avidity of all but one HCC intrahepatic lesion, reporting sensitivity of 97.0%, the majority of these cases showing intense FAPI avidity and a high lesion-to-background ratio [73]. 

FAPI was superior to FDG in detecting lesions, even in the milieu of existing liver cirrhosis, a low a-fetoprotein (AFP), multiple tumours, and non-serious microvessel invasion. Overall, FAPI also showed a significantly higher TBR than FDG [67,69,72]. FAPI was also found to be highly accurate (100%) compared to ceCTC, MRI, or a combination of both, reporting non-avidity in a pathologically benign lesion, which was seen as malignant on radiological scans, and showing avidity in a malignant lesion, which was undiscernible on MRI [68]. FAPI is also able to detect intrahepatic recurrence and residual HCC with high detection sensitivity of 100%; however, false positive uptake has also been noted in post-surgical/post-treatment inflammation, but this uptake generally has a typically diffuse pattern [70,72]. FAPI uptake was able to detect focal intrahepatic lesions, which MRI and follow up CT had confirmed to be post-treatment changes in two patients who had undergone TACE [72]). Siripongsatian et al. reported an advantage of ^68^Ga FAPI PET/MRI over FDG and MRI alone [72].

The size of the lesions did not contribute to the amount of FAPI uptake in HCC [68]. FAPI also detected more of small lesions (≤2 cm) than FDG [67].

Tumour differentiation did not contribute to the detection; hence, FAPI cannot characterise HCC lesions based on differentiation. It was better able to detect well to moderately differentiated lesions than FDG and demonstrated higher TBR than FDG in the poorly differentiated subtype in some studies [67,68,70]. Well and moderately differentiated HCC lesions show lower FAPI accumulation than poorly differentiated lesions [68].

FAPI also accumulates intensely in non-HCC liver cancers, such as cholangiocarcinoma, and metastases from malignancies, such as breast, colon, and gastric cancers. The TBR in HCC is lower than in cholangiocarcinoma and in metastatic hepatic lesions, and the intensity of uptake seemed higher in cholangiocarcinoma [68].

The accuracy of ^18^F FAPI in the differentiating of benign from malignant focal liver lesions was reported to be 83.8% in patients with suspicious non-FDG avid liver lesions [73]. Some benign lesions, such as cavernous haemangiomas, angiomyolipomas, inflammation, and FNH, may affect the specificity of FAPI [67,70,73]. FAPI is expressed in activated fibroblasts at wound healing/inflammatory sites, which may explain the uptake seen in inflammation [71]. In contrast, Shi et al. reported negligible intrahepatic uptake in benign lesions, suggesting the possibility of this tracer in the characterisation of intrahepatic lesions [69]. Dynamic FAPI PET imaging has been suggested as a potential method allowing for precise determination among HCC, non-HCC lesions, and healthy regions by showing significant differences in their kinetic modelling when evaluating a reversible two-tissue compartment model using both the arterial and venous input functions [74].

#### 4.5.2. Metastases

**Figure 3:**
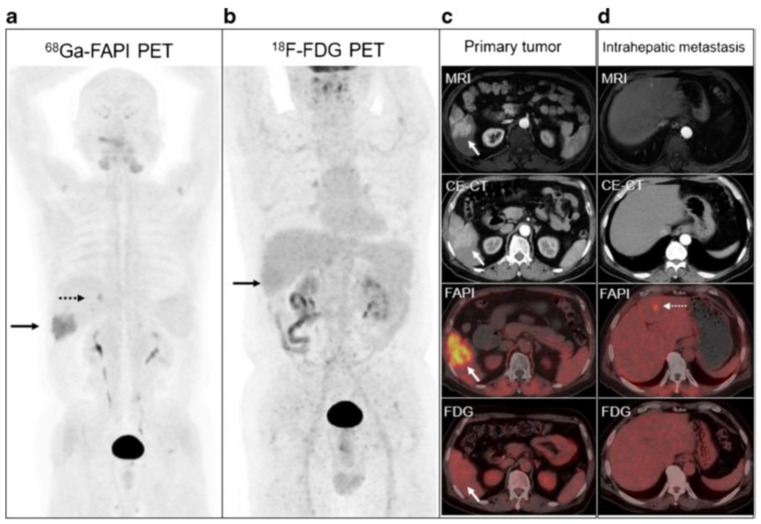
Patient with hepatitis B virus infection for initial staging of moderately differentiated HCC. ^68^Ga FAPI-04 (**a**), [^18^F]-FDG PET/CT (**b**), and MRI and contrast-enhanced CT (CE-CT) (**c**) concordantly showed the primary lesion at the right lower lobe (solid arrows). An extra 0.9-cm metastasis at the left internal lobe (dotted arrows) confirmed on ^68^Ga FAPI-04 PET/CT and liver MRI was not shown on CE-CT or [^18^F]-FDG PET/CT (**d**). (images reproduced with permission from [70]).

Wang et al. reported intense uptake in a poorly differentiated lymph node metastasis, as well as in a small peritoneal metastasis not detected by FDG [67]. Guo et al. also reported peritoneal metastases not evident on FDG imaging (Figure 3), possibly due to the fibrotic response elicited by tumour invasion of the peritoneal tissue [70]. FDG further missed the bone and brain lesions detected on FAPI imaging [70,72]. The uptake-positive rate of regional lymph node involvement was 100%, and these cases were detected with higher sensitivity and TBR than with FDG [72]. Further visceral and lymph node metastases have been detected with FAPI and overall with more intense uptake than FDG with the exception of lung metastases [70]. In comparison to conventional imaging, FAPI was able to upstage patients and contribute to a change in management by detecting new tumour lesions in 30%, modifying the BCLC stage in 10%, and adjusting treatment allocation in 10% of patients [70]. Major treatment changes have been reported, such as recommendations of additional systemic treatment when no tumours had been detected on conventional guideline compatible imaging or local ablation recommended when FAPI detected bifocal local recurrence or when occult recurrent HCC had been shown on conventional imaging [75].

FAPI uptake in other extrahepatic benign lesions, such as thyroid adenomas, pulmonary infections, and inflammatory granulomas, was noted [70].

### 4.6. Hypoxia Imaging

One of the mechanisms of action of TACE, T drug-eluting bead TACE, small-particle transcatheter arterial embolisation (TAE), yttrium 90 (^90^Y) radioembolisation TACE, and TACE within the tumour microenvironment is thought to be partly due to the induction of hypoxia. Shah et al. hypothesised that hypoxia imaging before and after embolisation therapy for hepatocellular carcinoma may help to assess response earlier than conventional radiological imaging and also help to predict short-term recurrence. 

The hypoxia tracer ^18^F-misonidazole (^18^F MISO) and its metabolites enter cells by passive diffusion, and they undergo reduction but no re-oxidation in hypoxic conditions and therefore are trapped in the cell. In these patients, FMISO was deemed to be undesirable due to its low target-to-background ratio in the liver with a median tumour-to-liver ratio (TLR) of 0.97 before treatment and 0.85 post-embolisation. No further studies were found using this PET probe in HCC [76].

### 4.7. Imaging in HCC Treated with SIRT

Intra-arterially directed therapies (IADTs) represent a group of loco-regional approaches based on the direct administration of a cytotoxic or embolic agent into HCC through its arterial feeders. IADTs play a crucial role for the clinical management of non-resectable HCC [77]. Among the different IADTs, SIRT consists of the intra-arterial delivery of microspheres labelled with Y-90 (resin or glass, Y-90 microspheres) or Ho-166 (poly-L-lactic-acid Ho-166 microspheres). 

Yttrium-90 (Y-90) is a pure β-emitter with a half-life of 64.2 h and with mean energy and tissue penetration of 0.937 MeV and 2.5 mm, respectively. Although these characteristics make it a suitable radionuclide for SIRT for HCC, and it has yielded favourable outcomes in well selected patients, it is not regarded as an imaging agent [78,79]. However, Y-90 microsphere imaging post-administration in the liver, using either the secondary bremsstrahlung radiation produced by the beta particles for single photon emission computed tomography (SPECT) or using Y-90 PET imaging, is possible because, as Y-90 decays, through internal pair production, it produces a positron and electron pair every 32 in one million decays, thus allowing for imaging with PET [80,81,82]. 

Post-treatment Y-90 PET imaging showed reasonable image quality when using time of flight (TOF) and Silicon photomultiplier-based PET scanners [81,82]. The images are useful for the assessment of treatment success by evaluation of the tracer accumulation and dosimetry in the targeted tumours; predicting complications, such as radioembolisation-induced liver disease; and determining the dose for subsequent treatments when needed [83].

In the determination of absorbed dose in HCC liver lesions and normal parenchyma, Y-90 PET was noted to show no significant difference compared with the conventionally used Tc-99m macroaggregated albumin (MAA) SPECT for resin microspheres. There was a trend towards a higher tumour absorbed dose noted with glass microspheres when using post-treatment Y-90 PET compared to pre-treatment Tc-99m MAA [82]. 

SIRT holds a place of particular relevance in HCC clinical work-ups since it combines embolisation and radiation therapy in a unique approach. SIRT can induce treatment-related changes in both HCC lesions and non-target hepatic parenchyma, making early response assessment by CT or MRI particularly challenging [84]. In this regard, both FDG and ^18^F choline have been applied for prognostication and response assessment in HCC patients submitted to SIRT, with interesting results. 

**Figure 4:**
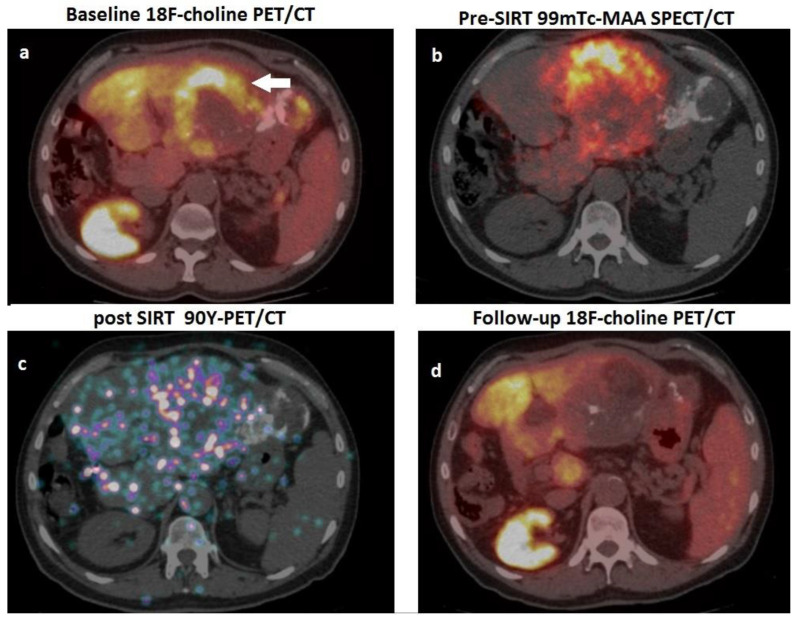
A 62-year-old man, affected by HCV-related cirrhosis, presenting a gross HCC of the left hepatic lobe, previously submitted to surgery (moderately differentiated HCC, pT2), subsequently relapsed and was treated with TACE. One year after TACE, he was referred to our department due to relapse and increased value of AFP (2357 ng/mL) for possible treatment with ^90^Y-microspheres. (**a**) Pre-treatment PET/CT scan with 18F-choline showed a hemicircular rim of increased tracer uptake (white arrow) within the HCC lesion in the left hepatic lobe (note the hyperdense material in the neighbouring parenchyma due to the previously performed TACE). (**b**) The patient was submitted to scintigraphy with albumin 99mTc-macroaggregates (i.e., PRE-SIRT), and coregistered SPECT-CT images showed an optimal match with respect to the location of the metabolically active lesion detected by PET/CT; (**c**) After selective catheterisation of the arterial feeder of the HCC lesion, 1.8 Gbq of 90Y-resin microspheres were administered; PET/CT acquired on 90Y-positron emission showed microspheres’ accumulation within the hepatic lesions; (**d**) 18F-choline PET/CT performed eight weeks after SIRT showed an almost complete metabolic response, consistent with a dramatic drop in AFP values (i.e., 50 ng/mL).

Concerning the applications of FDG PET/CT, it has been reported that changes in total lesion glycolysis (∆TLG) measured 1 month after SIRT are associated with a trend towards longer survival in poorly differentiated HCC with evidence of portal vein invasion (PVI) treated with Y-90 resin microspheres [85]. Regarding radiolabelled choline, in 24 patients with locally advanced HCC and initially increased AFP levels, Hartenbach et al. employed PET/CT with ^18^F fluoroethylcholine to predict response to SIRT: in these cases, reduction in SUV max and in tumour-to-background ratio after Y-90 administration showed the highest predictive value [57]. Few papers have investigated the potential of dual tracer PET/CT with FDG/ ^18^F choline in HCC patients submitted to SIRT. Reizine and coworkers evaluated 37 HCC patients submitted to both FDG and ^18^F choline PET/CT scans before Y-90 therapy: 28 subjects exhibited FDG-positive lesions, while nine patients were 18F-choline positive. The authors found early metabolic response, assessed by ^18^F choline or FDG PET/CT PET/CT at 4–8 weeks post-treatment, to be 100% sensitive and 100% specific for the prediction of 6-month radiological response evaluated according to mRECIST [86]. Very recently, a retrospective real-world study including 21 HCC patients submitted to SIRT with Y-90 microspheres highlighted the clinical usefulness of response assessment performed with ^18^F choline or FDG PET/CT at 8 weeks after SIRT: PET/CT influenced clinical management in 10 cases (47.6%), providing indications for a second Y-90 administration targeting metabolically active HCC remnants or leading to PET-guided radiotherapy of metastatic nodes [87]. Figure 4 represents a case from the authors’ series, depicting the clinical usefulness of PET/CT with ^18^F choline for response assessment after SIRT.

## 5. Conclusions

Several molecular probes have been shown to have clinical potential in HCC lesion detection, characterisation, staging, and recurrence. Approximately one-third of HCCs do not metabolise ^18^F FDG, mainly showing low sensitivity in the detection of low-grade HCC. PET radiopharmaceuticals, such as ^68^Ga PSMA, ^18^F/^11^C-choline, and ^11^C-acetate, appear to play a complementary role in assessing these non-FDG avid tumours. These radiotracers, including FAPI and PSMA, commonly surpassed FDG in sensitivity, intensity of uptake, and TBR. PET probes in HCC are changing the landscape of HCC management and are noted to improve the accuracy of conventional radiological imaging in this malignancy.

## Figures and Tables

**Table 1:** Summary of manuscripts comparing FDG and other PET probes for imaging HCC. * Only patients with HCC were selected; ^ǂ^ Only calculated for extrahepatic metastases; Adapted from Filipi et al. [49].

References	Year of Publication	Type of Study	Metabolic Probes	Patients (N)	Sensitivity (%)	Specificity (%)	Comment
Ho et al. [5]	2003	Single centre, prospective	FDG, ^11^C-acetate	57	FDG: 47.3 ^11^C-acetate: 87.3	Not available	Evaluation of the characteristics of ^11^C-acetate and FDG metabolism in HCC and other liver masses
Park et al. [33]	2008	Single centre, prospective	FDG, ^11^C-acetate	112	FDG: 60.9 ^11^C-acetate: 75.4	Not available	Biopsy-based analysis demonstrated lower sensitivity of FDG PET for primary HCC compared with ^11^C-acetate
Cheung et al. [63]	2013	Single centre, retrospective	FDG, ^11^C-acetate	43	FDG: 32.8 ^11^C-acetate: 93.1	FDG: 100 ^11^C-acetate:100	Sensitivity and specificity of dual-tracer PET for liver transplantation selection were significantly higher than those of contrast CT
Li S et al. [64]	2017	Single centre prospective	FDG, ^11^C-acetate	22	FDG: 45 ^11^C-acetate: 68	* Not available	Assessment of the response in HCC treated with TACE plus bevacizumab
Yamamoto et al. [48]	2008	Single centre, retrospective	FDG, ^11^C-choline	12	FDG: 63 ^11^C-choline: 50	* Not available	^11^C-choline is a potential tracer to complement FDG in detection of HCC lesions
Wu et al. [56]	2011	Single centre prospective	FDG, ^11^C-choline	76	FDG: 63.1 ^11^C-choline: 71.4	^ǂ^ FDG: 94.8 ^11^C-choline: Not available	The dual-tracer modality improved the diagnostic sensitivity of FDG PET
Talbot et al. [27]	2010	Single centre, prospective	FDG, FCH	81	FDG: 88 FCH: 68	FDG: 94 FCH: 47	FCH proved useful to detect HCC, but dual tracer PET resulted in the best option
Castilla-Lièvre et al. [55]	2016	Single centre, prospective	FDG, ^11^C-choline	33	FDG: ^11^C-choline:	FDG: ^11^C-choline	The combined use of ^11^C-choline and FDG detected HCC with high sensitivity
Chalaye J et al [65].	2018	Multicentre, retrospective	FDG, FCH	177	Not available	Not available	Dual-tracer PET-CT is able to disclose lesions not detected by conventional imaging
Kesler [43]	2019	Prospective pilot study	PSMA, FDG	7	Not available	Not available	^68^Ga PSMA was superior to ^18^F -FDG for imaging patients with HCC and also detected unexpected extrahepatic metastases
Kuyumcu S et al. [66]	2019	Single centre, prospective Patients for restaging	PSMA, FDG	19	Not available	Not available	No significant difference between PSMA and FDG in advance HCC. Regenerative nodules did not accumulate PSMA. PSMA imaging detected metastases not seen on CT, including in bone marrow, adrenal, and peritoneum.
Gündoğan C et al. [46]	2021	Single centre, prospective Patients for restaging	PSMA, FDG	14	Not available	Not available	^68^Ga PSMA is superior to ^18^F -FDG PET/CT in diagnosis and staging of HCC and is superior to MRI in demonstrating extrahepatic involvement
Wang et al. [67]	2021	Prospective—post-TACE and post-surgery	FAPI, FDG	29	FDG: 57.1 FAPI: 85.7	Not available	FAPI sensitivity is superior to FDG. FAPI is sensitive in staging patients, including those with cirrhosis, low AFP, multiple tumours, and microvessel invasion. It also detected more of the small lesions, including in extrahepatic disease involvement.
Shi X et al., pilot [68]	2021	Prospective pilot study—detection in hepatic cancers not specific for HCC	FAPI, FDG	20 (14 with HCC)	FDG: 58.8 FAPI: 100	FDG: 100 FAPI: 100	FAPI was more sensitive in detecting primary hepatic cancer, with 100% sensitivity and specificity. It resulted in higher TBR than FDG
Shi et al. [69]	2021	Retrospective, single-centre	FAPI, FDG	25 (17 with HCC)	Not available	Not available	FAPI was 100% sensitive, while FDG was only 58%. FAPI with dedicated MRI was superior in detecting hepatic malignancy than FDG and MRI alone
Guo et al. [70]	2021	Retrospective, single-centre	FAPI, FDG	34 (20 with HCC)	FDG: 65 FAPI: 96	Not available	Detection sensitivity with FAPI was 100% in upstaged patients, leading to a change in management in up to 30%
